# Modern rain-isotope data from Indian island and the mainland on the daily scale for the summer monsoon season

**DOI:** 10.1016/j.dib.2019.103793

**Published:** 2019-03-01

**Authors:** Nitesh Sinha, S. Chakraborty, P.M. Mohan

**Affiliations:** a Centre for Climate Change Research, Indian Institute of Tropical Meteorology, Pune, India; b Department of Atmospheric and Space Science, Savitribai Phule Pune University, Pune, India; c Department of Ocean Studies and Marine Biology, Pondicherry University, Port Blair, Andaman and Nicobar Islands, India

**Keywords:** Stable rain isotopes, Indian summer monsoon, Bay of Bengal

## Abstract

The atmospheric stable isotopes in rainwater are measured as the ratio of heavy to the lighter (e.g., ^18^O/^16^O). This data article describes two datasets of daily rain isotopic variations during the Indian Summer Monsoon (ISM) season. Firstly, an island site (namely Port Blair) dataset for four years (2012–2015) and another dataset of Indian mainland sites (namely Nagpur, Tezpur, and Kolkata) for the year 2015. Port Blair is strategically situated in the Bay of Bengal (BoB), which is known to be a significant moisture source to ISM rainfall over the Indian landmass. On the other hand, sites in the mainland are receivers of the BoB moisture. This dataset includes 1030 isotopic analyses made on daily water samples as well as rainfall amount (24 hours) during ISM.

The data is related to “Isotopic investigation of the moisture transport processes over the Bay of Bengal” [1].

**Table:** Specifications table

Subject area	Atmospheric Science
More specific subject area	Isotope Hydrology
Type of data	Table
How data was acquired	LGR's Liquid Water Isotope Analyzer (LWIA), Model No.: TIWA-45-EP
Data format	Analyzed
Experimental factors	Rainwater sampling were done on a daily scale, i.e., once in 24 hours for each rainy day
Experimental features	Water samples were collected using standard rain gauge and transferred to tightly sealed 30 mL bottles and stored at room temperature (cool and dry) prior to analysis.
Data source location	Sample collection: India {Port Blair (11.66^o^N, 92.73^o^E); Nagpur (21.15°N, 79.09°E); Kolkata (22.56°N, 88.36°E) Tezpur (26.63°N, 92.80°E)}. Samples were analyzed at Indian Institute of Tropical Meteorology, Pune
Data accessibility	The data are available with this article
Related research article	Nitesh Sinha, S. Chakraborty, Rajib Chattopadhyay, B.N. Goswami, P.M. Mohan, Dipak K. Parua, Dipankar Sarma, Amey Datye, S. Sengupta, Subir Bera, K.K. Baruah, Isotopic investigation of the moisture transport processes over the Bay of Bengal, Journal of Hydrology X, 2, 2019, https://doi.org/10.1016/j.hydroa.2019.100021[1].

**Table:** 

**Value of the data** • These datasets are useful to trace the moisture source regions and understand the moisture transport process over the Indian subcontinent [1], [2], [3]. • Due to the distinct isotopic characteristics, the rainwater samples from source and receiver regions provide a means to determine the contributions of land and ocean-derived moisture [4]. • Give new insights into amount effect variability [2], which should be taken into account for future paleoclimate studies from a proxy [5], [6], [7], [8]. • The isotopic data offer new insight into water cycling processes, spatial and temporal variability in surface water balance.

## 1 Data

The rainwater samples from a few sites in the Indian subcontinent were collected, mainly during the northern hemispheric summer monsoon months. Daily rainwater was collected at Port Blair (2012–2015) once in a day at 08:30 local time (UTC+05:30). Similarly, rainwater samples were also collected at Indian mainland sites, such as Nagpur, Kolkata, and Tezpur for the year 2015. The sampling details and isotope ratio measurements for Port Blair, Andaman Islands (Table 1.1) and mainland sites (Table 1.2) are presented.

**Table 1.1:** Port Blair dataset (2012–15) includes date of sampling, isotopic ratios of oxygen (δ^18^O) and hydrogen (δD) in rainwater (permil, ‰), and 24 hours rainfall amount in mm/day (mm/d). For the years 2012–2014, daily gridded rainfall data of the Tropical Rain Measuring Mission (TRMM 3b42 v2; 0.25 × 0.25 [10]) was used and the amount of the rain collected in 2015 was obtained in the field (observation).

Port Blair	TRMM			Date	TRMM			Date	TRMM			Date	Observation		
Date	δ^18^O (‰)	δD (‰)	Rain (mm/d)		δ^18^O (‰)	δD (‰)	Rain (mm/d)		δ^18^O (‰)	δD (‰)	Rain (mm/d)		δ^18^O (‰)	δD (‰)	Rain (mm/d)
**02-05-12**	−3.05	−8.73	65.3	**02-05-13**	-3.34	-21.46	1.5	**06-05-14**	−3.71	−10.02	0.0	**24-05-15**	−3.14	−12.29	119.3
**03-05-12**	−6.11	−31.43	28.3	**11-05-13**	−1.75	−11.27	19.1	**07-05-14**	−1.91	−3.60	24.7	**25-05-15**	−3.87	−17.21	13.1
**04-05-12**	−3.93	−11.34	2.0	**13-05-13**	−2.07	−11.40	5.8	**08-05-14**	−2.99	−8.00	6.2	**28-05-15**	−2.96	−12.81	9.8
**05-05-12**	−3.42	−7.37	60.2	**16-05-13**	−2.70	−13.43	41.6	**09-05-14**	−1.11	−6.40	0.4	**29-05-15**	−2.23	−5.97	11.4
**06-05-12**	−1.38	6.99	130.5	**19-05-13**	−3.60	−21.66	15.5	**10-05-14**	−2.22	−8.01	0.0	**30-05-15**	−2.93	−11.18	9.6
**09-05-12**	−2.01	7.15	0.0	**20-05-13**	−3.66	−21.29	45.8	**11-05-14**	−4.41	−23.33	0.3	**31-05-15**	−3.56	−15.29	9.6
**12-05-12**	−1.23	8.09	0.0	**21-05-13**	−3.94	−21.75	63.2	**12-05-14**	−3.67	−19.83	2.0	**01-06-15**	−2.51	−7.74	3.1
**17-05-12**	−2.83	−3.01	5.6	**22-05-13**	−4.03	−21.83	0.3	**13-05-14**	−3.49	−19.46	40.7	**02-06-15**	−2.51	−10.70	9.2
**19-05-12**	−0.87	6.54	22.6	**23-05-13**	−3.02	−21.03	5.6	**14-05-14**	−3.56	−19.51	14.3	**04-06-15**	−1.55	−4.46	8.8
**20-05-12**	−1.21	5.74	41.7	**26-05-13**	−3.12	−20.98	67.2	**16-05-14**	−6.12	−42.20	0.1	**05-06-15**	−1.94	−8.46	2.7
**22-05-12**	−0.74	7.63	0.7	**06-06-13**	−3.19	−19.66	7.2	**17-05-14**	−3.69	−19.86	5.0	**12-06-15**	−2.24	−10.20	50.4
**24-05-12**	−2.54	−5.70	3.2	**11-06-13**	−3.97	−21.29	60.7	**20-05-14**	−3.74	−19.82	20.4	**13-06-15**	−1.48	−5.13	1.1
**26-05-12**	−5.04	−24.49	14.7	**12-06-13**	0.72	5.82	21.5	**22-05-14**	2.56	10.71	4.2	**15-06-15**	−1.21	−6.22	0.0
**28-05-12**	−2.11	−2.74	14.8	**13-06-13**	−0.02	5.61	16.7	**24-05-14**	−4.06	−16.75	0.0	**16-06-15**	−2.47	−14.28	35.1
**31-05-12**	−0.53	5.63	9.0	**14-06-13**	−1.01	−0.85	6.7	**25-05-14**	−4.05	−16.78	27.1	**17-06-15**	−4.51	−29.98	40.5
**04-06-12**	0.38	8.95	1.7	**15-06-13**	−0.80	−0.89	72.2	**27-05-14**	−1.57	−13.73	0.0	**18-06-15**	−4.63	−31.46	36.3
**06-06-12**	−3.84	−22.51	28.9	**16-06-13**	−1.38	−1.06	9.3	**28-05-14**	−5.75	−34.04	1.0	**19-06-15**	−2.14	−13.29	28.7
**07-06-12**	−2.80	−8.78	2.5	**18-06-13**	−3.70	−19.99	34.7	**31-05-14**	−3.82	−18.25	0.0	**20-06-15**	−3.05	−18.61	38.4
**09-06-12**	−5.25	−29.71	76.6	**19-06-13**	−3.90	−20.54	86.6	**03-06-14**	−4.76	−27.14	0.0	**21-06-15**	−0.15	1.82	4.1
**10-06-12**	−5.51	−28.75	0.8	**20-06-13**	−3.65	−18.90	30.6	**05-06-14**	−4.45	−21.19	1.6	**22-06-15**	0.04	5.01	0.2
**11-06-12**	−3.47	−15.14	28.8	**21-06-13**	0.86	4.19	5.1	**06-06-14**	−8.37	−50.72	12.3	**23-06-15**	−0.28	3.08	4.7
**12-06-12**	−2.32	−3.52	72.8	**22-06-13**	−1.82	−9.09	34.1	**07-06-14**	−5.07	−32.35	143.9	**24-06-15**	−0.93	1.93	8.8
**13-06-12**	−0.50	5.73	4.3	**23-06-13**	−2.41	−9.60	18.1	**08-06-14**	−2.49	−16.43	8.3	**25-06-15**	−1.21	1.38	6.3
**15-06-12**	−1.27	2.41	7.7	**24-06-13**	3.05	12.22	0.0	**09-06-14**	−2.68	−18.26	4.0	**26-06-15**	−0.95	2.25	6.5
**25-06-12**	−2.12	−5.64	11.8	**25-06-13**	−3.81	−22.14	0.0	**10-06-14**	−2.52	−8.85	16.2	**27-06-15**	−1.97	−2.14	20.1
**28-06-12**	−0.59	4.41	0.2	**27-06-13**	−3.28	−17.64	5.6	**11-06-14**	0.02	0.85	7.3	**28-06-15**	−3.78	−14.31	59.7
**07-07-12**	−1.20	1.11	34.6	**30-06-13**	−3.30	−17.46	5.5	**12-06-14**	−3.17	−13.53	1.5	**29-06-15**	−2.20	−8.21	27.2
**09-07-12**	−2.07	−3.38	55.2	**02-07-13**	−1.65	−5.37	1.9	**14-06-14**	−2.80	−11.98	20.8	**01-07-15**	−2.36	−8.47	8.2
**11-07-12**	−2.28	−5.53	8.0	**03-07-13**	−1.71	−4.58	33.8	**15-06-14**	−4.72	−26.49	0.8	**02-07-15**	−2.88	−11.14	22.8
**12-07-12**	−4.13	−22.73	8.4	**05-07-13**	−2.44	−14.62	0.7	**16-06-14**	−4.27	−19.59	104.5	**03-07-15**	−1.48	0.33	4.0
**19-07-12**	−1.98	−9.70	12.4	**07-07-13**	−2.51	−14.63	12.4	**22-06-14**	−0.78	3.49	0.0	**04-07-15**	−0.85	4.44	6.1
**25-07-12**	−0.56	1.58	15.7	**09-07-13**	−5.06	−27.56	21.9	**23-06-14**	−1.28	0.56	0.5	**05-07-15**	−0.82	5.04	2.4
**27-07-12**	−0.93	−0.51	34.0	**10-07-13**	−4.26	−21.34	64.2	**26-06-14**	0.09	8.51	6.8	**06-07-15**	−0.63	5.62	2.7
**29-07-12**	−0.84	1.09	16.2	**11-07-13**	−4.19	−21.34	34.2	**01-07-14**	−1.12	0.26	0.6	**10-07-15**	−0.73	4.89	0.8
**31-07-12**	−0.85	3.50	0.2	**12-07-13**	−4.04	−21.93	1.4	**02-07-14**	−2.40	−8.42	0.8	**11-07-15**	0.02	7.59	9.3
**11-08-12**	−2.82	−10.39	1.8	**14-07-13**	−5.40	−28.89	84.1	**03-07-14**	−1.16	−1.68	11.2	**12-07-15**	−0.98	−0.03	5.6
**14-08-12**	−2.00	−4.08	1.9	**15-07-13**	−5.23	−29.60	45.9	**06-07-14**	−3.75	−20.71	0.6	**13-07-15**	−0.25	3.18	12.1
**16-08-12**	−2.24	−6.47	0.0	**16-07-13**	−1.74	0.11	36.1	**08-07-14**	−5.01	−33.72	43.8	**14-07-15**	−0.87	0.60	78.3
**17-08-12**	−2.35	−9.95	7.1	**17-07-13**	−2.24	−10.52	8.8	**09-07-14**	−3.38	−21.27	74.6	**15-07-15**	−0.37	4.42	4.7
**30-08-12**	−6.13	−34.43	16.1	**18-07-13**	−1.52	−2.36	39.5	**10-07-14**	−5.07	−32.59	107.9	**16-07-15**	−0.91	0.61	9.7
**03-09-12**	−6.34	−34.83	6.0	**19-07-13**	−2.76	−10.22	7.2	**11-07-14**	−3.38	−18.16	15.6	**17-07-15**	−0.46	1.20	5.1
**04-09-12**	−6.29	−34.64	2.7	**21-07-13**	−1.74	−3.03	5.4	**12-07-14**	−1.29	0.43	13.0	**18-07-15**	−0.21	3.84	1.2
**05-09-12**	−6.45	−35.08	8.4	**22-07-13**	−1.61	−3.18	27.0	**13-07-14**	−1.04	2.26	71.6	**20-07-15**	−2.75	−13.02	52.2
**06-09-12**	−6.53	−35.00	0.1	**23-07-13**	−4.18	−22.43	40.7	**14-07-14**	−2.62	−7.62	4.4	**21-07-15**	−3.39	−18.50	26.4
**07-09-12**	−6.39	−34.69	179.1	**25-07-13**	1.24	8.82	2.2	**15-07-14**	−4.99	−26.92	39.6	**22-07-15**	−3.14	−14.15	5.6
**08-09-12**	−6.55	−34.93	9.6	**01-08-13**	−0.69	1.81	1.9	**16-07-14**	−2.51	−6.82	30.8	**23-07-15**	−4.47	−22.94	16.0
**09-09-12**	−6.54	−34.65	35.9	**02-08-13**	−0.33	1.88	17.3	**17-07-14**	−0.22	6.88	0.9	**24-07-15**	−4.02	−18.75	9.2
**10-09-12**	−6.33	−34.65	117.8	**03-08-13**	−2.33	−6.51	20.4	**18-07-14**	−0.90	5.02	6.1	**26-07-15**	−3.32	−11.78	1.0
**11-09-12**	−6.11	−41.80	4.4	**04-08-13**	−0.73	−0.50	8.4	**19-07-14**	−2.49	−9.66	8.2	**27-07-15**	−1.92	0.52	0.0
**12-09-12**	−7.22	−42.17	11.7	**05-08-13**	−0.41	−0.79	0.1	**22-07-14**	−1.42	2.78	1.7	**28-07-15**	−2.35	−0.51	0.9
**13-09-12**	−6.59	−40.90	25.5	**10-08-13**	−3.13	−23.24	25.3	**23-07-14**	−2.63	−10.28	120.7	**29-07-15**	−3.27	−2.82	0.8
**14-09-12**	−6.15	−39.60	42.4	**11-08-13**	−3.75	−22.57	3.7	**24-07-14**	−2.20	−10.16	18.2	**30-07-15**	−4.24	−7.78	3.2
**15-09-12**	−6.68	−41.73	134.4	**12-08-13**	−3.71	−22.70	21.8	**25-07-14**	−1.94	−9.10	13.6	**02-08-15**	−3.78	−2.11	9.5
**16-09-12**	−6.04	−37.11	99.3	**13-08-13**	−2.67	−11.61	25.9	**26-07-14**	−1.75	−8.55	53.8	**03-08-15**	0.20	1.56	14.9
**17-09-12**	−6.88	−41.81	5.0	**14-08-13**	−7.88	−51.64	16.3	**27-07-14**	−2.92	−18.96	37.9	**04-08-15**	−1.13	−3.82	30.9
**18-09-12**	−7.10	−40.41	73.1	**20-08-13**	−0.83	2.23	10.0	**03-08-14**	1.39	6.05	0.1	**05-08-15**	−2.39	−13.06	29.1
**20-09-12**	−5.37	−26.42	12.2	**22-08-13**	−0.94	2.12	9.6	**06-08-14**	−3.17	−17.15	40.0	**07-08-15**	−3.37	−16.37	77.3
**21-09-12**	−4.10	−15.91	17.8	**29-08-13**	−6.35	−40.10	4.8	**07-08-14**	−4.20	−25.71	39.3	**08-08-15**	−1.95	−3.62	37.8
**22-09-12**	−2.52	−14.63	0.3	**01-09-13**	−6.84	−43.11	28.7	**08-08-14**	−6.25	−40.45	31.7	**09-08-15**	−1.94	−3.21	16.0
**23-09-12**	−3.33	−14.72	12.7	**02-09-13**	−6.67	−40.21	6.6	**09-08-14**	−0.50	−2.49	3.5	**10-08-15**	−1.79	0.12	3.3
**24-09-12**	−3.21	−14.47	20.8	**06-09-13**	−6.11	−41.59	6.0	**11-08-14**	−0.32	2.42	0.7	**11-08-15**	−2.63	−5.67	1.7
**25-09-12**	−4.06	−16.04	5.4	**08-09-13**	−5.96	−41.04	8.6	**12-08-14**	−1.09	−6.64	4.2	**12-08-15**	−1.31	1.90	2.4
**26-09-12**	−3.63	−12.50	6.4	**11-09-13**	−8.75	−57.11	14.0	**13-08-14**	−1.82	−8.60	12.5	**13-08-15**	−1.82	−0.40	6.5
**27-09-12**	−5.52	−30.90	2.6	**12-09-13**	−8.49	−56.85	120.1	**14-08-14**	1.02	2.31	2.4	**14-08-15**	−1.30	2.60	0.7
**28-09-12**	−6.82	−38.48	15.0	**13-09-13**	−3.19	−11.11	11.8	**15-08-14**	−2.40	−10.01	1.0	**16-08-15**	−1.30	0.17	0.8
**29-09-12**	−3.11	−11.53	21.2	**14-09-13**	−2.76	−10.05	24.1	**18-08-14**	−1.52	−10.02	1.0	**19-08-15**	−3.33	−15.82	0.0
**30-09-12**	−2.05	−10.10	4.9	**15-09-13**	−2.25	−9.63	7.2	**20-08-14**	−5.93	−37.29	0.0	**20-08-15**	−2.26	−11.97	0.6
**01-10-12**	−0.84	−2.93	30.9	**16-09-13**	−2.39	−9.18	0.1	**22-08-14**	−2.73	−10.91	1.5	**21-08-15**	−2.31	−12.40	1.6
**02-10-12**	−0.82	0.73	0.4	**17-09-13**	−2.37	−9.69	12.1	**25-08-14**	−2.27	−9.57	0.0	**22-08-15**	−5.05	−31.31	46.7
**03-10-12**	0.70	3.43	1.8	**18-09-13**	−2.55	−9.23	4.2	**26-08-14**	−5.89	−37.85	2.1	**23-08-15**	−5.55	−38.20	18.0
**06-10-12**	−3.11	−16.03	3.6	**19-09-13**	−2.49	−9.97	18.2	**27-08-14**	−3.53	−13.95	31.8	**24-08-15**	−2.95	−14.29	15.9
**07-10-12**	−3.56	−15.23	8.5	**20-09-13**	−3.11	−10.89	16.5	**29-08-14**	−0.41	0.32	0.3	**25-08-15**	−2.50	−10.46	29.2
**09-10-12**	−4.80	−26.38	6.5	**21-09-13**	−2.48	−10.57	21.6	**30-08-14**	−2.35	−4.98	0.8	**26-08-15**	−3.22	−12.54	78.5
**12-10-12**	−2.40	−9.21	0.3	**22-09-13**	−3.10	−11.23	11.6	**04-09-14**	−2.22	−11.44	2.0	**27-08-15**	−4.73	−22.65	32.5
**14-10-12**	−3.24	−14.65	0.6	**23-09-13**	−0.15	3.79	11.7	**05-09-14**	−2.05	−11.44	5.2	**28-08-15**	−2.84	−7.76	17.7
**15-10-12**	−3.27	−13.46	4.6	**24-09-13**	−0.86	3.34	24.0	**06-09-14**	−2.35	−11.18	60.2	**29-08-15**	−2.87	−8.55	3.3
**16-10-12**	−2.96	−13.14	8.9	**25-09-13**	−0.91	3.37	0.9	**07-09-14**	−2.23	−10.88	25.7	**30-08-15**	−1.60	−2.38	2.3
**17-10-12**	−2.15	−12.87	0.0	**26-09-13**	−0.24	3.66	10.3	**08-09-14**	−2.25	−11.02	4.5	**31-08-15**	−1.16	−2.93	1.9
**18-10-12**	−3.49	−14.48	0.0	**28-09-13**	−0.43	3.83	11.5	**09-09-14**	−2.55	−6.64	7.1	**01-09-15**	−3.68	−28.42	11.6
**26-10-12**	−5.04	−28.06	18.4	**29-09-13**	−5.06	−23.58	4.0	**10-09-14**	−3.56	−16.90	90.5	**02-09-15**	−3.34	−23.87	8.9
				**30-09-13**	−5.19	−23.50	0.0	**11-09-14**	2.62	13.26	2.4	**03-09-15**	−5.41	−39.98	11.6
				**04-10-13**	−5.34	−24.43	55.2	**12-09-14**	−3.23	−15.25	0.0	**04-09-15**	−4.52	−30.52	12.2
				**05-10-13**	−4.74	−22.72	0.7	**14-09-14**	−1.69	−6.06	19.0	**05-09-15**	−6.03	−38.99	17.1
				**06-10-13**	−4.36	−23.28	0.0	**15-09-14**	0.75	0.77	15.4	**06-09-15**	−7.55	−55.33	8.4
				**07-10-13**	−4.31	−23.55	91.1	**16-09-14**	0.45	0.56	14.4	**07-09-15**	−4.74	−33.66	0.2
				**09-10-13**	−5.80	−31.84	9.8	**30-09-14**	−6.80	−42.22	0.0	**09-09-15**	−4.40	−27.93	12.9
				**10-10-13**	−5.37	−30.76	9.9	**03-10-14**	−6.55	−41.98	0.1	**10-09-15**	−7.26	−52.46	14.4
				**13-11-13**	−10.47	−68.15	0.0	**04-10-14**	−6.47	−43.65	124.6	**11-09-15**	−8.99	−65.68	29.0
				**23-11-13**	−10.42	−68.22	21.1	**06-10-14**	−6.22	−37.29	1.5	**12-09-15**	−6.34	−40.27	36.5
								**07-10-14**	−12.20	−89.20	43.3	**13-09-15**	−0.78	−3.79	12.9
								**09-10-14**	−2.59	−13.12	8.9	**14-09-15**	−1.45	−2.03	11.0
								**10-10-14**	−3.07	−14.69	16.1	**15-09-15**	−1.48	−3.87	0.6
								**13-10-14**	−1.81	−13.25	12.7	**16-09-15**	−0.62	−0.42	1.7
								**17-10-14**	−2.18	−16.28	3.0	**17-09-15**	−1.58	−2.66	30.5
								**26-10-14**	−6.40	−37.24	19.6	**18-09-15**	−7.40	−48.10	60.5
								**27-10-14**	−6.24	−41.64	31.5	**19-09-15**	−8.56	−55.33	105.0
								**28-10-14**	−4.11	−29.58	10.5	**26-09-15**	−4.69	−21.07	7.9
								**29-10-14**	−3.92	−28.93	1.7	**27-09-15**	−2.47	−10.60	1.6
								**30-10-14**	−2.67	−15.33	0.0	**28-09-15**	−3.88	−17.65	0.0
								**02-11-14**	−8.95	−53.73	3.9	**29-09-15**	−5.08	−35.21	20.6
								**03-11-14**	−7.76	−45.69	38.6	**30-09-15**	−4.19	−30.99	2.1
								**04-11-14**	−10.08	−66.09	16.1	**01-10-15**	−4.93	−34.89	0.3
								**05-11-14**	−7.13	−46.68	37.5	**02-10-15**	−4.21	−22.15	0.1
								**10-11-14**	−3.89	−22.63	2.8	**03-10-15**	−4.62	−27.02	33.9
								**11-11-14**	−9.39	−59.55	2.7	**04-10-15**	−6.49	−41.11	27.2
								**12-11-14**	0.47	−10.85	14.2	**05-10-15**	−6.10	−37.26	20.0
								**28-11-14**	−5.42	−27.45	2.7	**06-10-15**	−3.60	−16.57	5.2
								**29-11-14**	−5.24	−30.64	30.0	**07-10-15**	−2.98	−13.41	6.3

**Table 1.2:** Mainland dataset (2015) includes date of sampling, isotopic ratios of oxygen (δ^18^O) and hydrogen (δD) in rainwater (permil, ‰), and 24 hours rainfall amount in mm/day (mm/d). The daily gridded rainfall data of the Global Precipitation Measurement (GPM; 0.1 × 0.1 [11]) was used.

Kolkata				Nagpur				Tezpur			
Date	GPM			Date	GPM			Date	GPM		
	δ^18^O (‰)	δD (‰)	Rain (mm/d)		δ^18^O (‰)	δD (‰)	Rain (mm/d)		δ^18^O (‰)	δD (‰)	Rain (mm/d)
**25-04-15**	−2.00	3.38	2.9	**10-07-15**	0.65	10.52	8.3	**02-04-15**	−1.75	−4.54	7.9
**28-04-15**	−3.37	−2.82	7.6	**13-07-15**	1.89	18.82	0.2	**03-04-15**	−0.97	0.28	0.0
**02-05-15**	1.13	14.20	0.8	**20-07-15**	−1.22	−4.08	0.8	**04-04-15**	−1.44	−2.33	2.4
**16-05-15**	−4.52	−18.26	4.1	**22-07-15**	−5.00	−30.19	3.6	**05-04-15**	−1.13	−0.72	5.8
**20-05-15**	−2.20	−2.39	0.8	**23-07-15**	−8.17	−51.88	4.8	**08-04-15**	1.37	17.97	1.1
**20-06-15**	−8.09	−52.70	2.8	**24-07-15**	−7.25	−42.63	9.2	**21-04-15**	−1.80	0.23	6.8
**24-06-15**	−10.27	−70.99	5.0	**25-07-15**	−2.88	−14.91	0.0	**22-04-15**	−1.51	1.45	3.0
**28-06-15**	−6.09	−38.60	10.2	**28-07-15**	−0.78	5.97	0.6	**23-04-15**	−1.17	1.77	3.8
**30-06-15**	−0.86	0.98	2.1	**29-07-15**	−0.66	4.31	0.0	**25-04-15**	0.05	14.76	0.7
**02-07-15**	−3.80	−18.45	8.2	**04-08-15**	−11.03	−75.23	21.3	**29-04-15**	−1.02	6.57	4.5
**03-07-15**	0.40	14.58	2.6	**05-08-15**	−5.20	−31.59	0.1	**04-05-15**	1.88	24.42	0.5
**06-07-15**	−2.16	−5.71	0.9	**07-08-15**	−3.30	−13.67	1.2	**15-05-15**	1.86	24.25	5.4
**08-07-15**	−5.05	−32.27	13.4	**27-08-15**	0.35	11.40	1.4	**16-05-15**	0.66	12.63	0.3
**09-07-15**	−3.92	−25.96	47.4	**28-08-15**	−2.39	−10.74	6.9	**18-05-15**	−1.21	−0.73	1.7
**13-07-15**	−0.85	−2.79	1.5	**30-08-15**	−0.40	6.54	2.0	**22-05-15**	−0.81	1.06	2.6
**14-07-15**	−2.02	−6.77	0.0	**02-09-15**	−1.93	−7.45	0.0	**25-05-15**	0.81	15.37	4.7
**15-07-15**	−3.38	−20.95	23.0	**07-09-15**	1.13	16.40	3.5	**27-05-15**	1.60	26.34	0.4
**17-07-15**	−6.93	−52.44	9.6	**17-09-15**	−12.27	−87.42	15.0	**28-05-15**	0.93	20.57	4.0
**20-07-15**	−4.98	−40.19	8.8	**18-09-15**	−6.37	−39.29	0.4	**29-05-15**	0.56	18.45	0.4
**21-07-15**	−3.53	−27.90	1.4					**01-06-15**	−0.01	11.88	1.0
**22-07-15**	−3.64	−18.61	5.0					**02-06-15**	0.34	14.09	0.5
**23-07-15**	−5.34	−36.00	2.3					**03-06-15**	0.21	15.15	2.1
**24-07-15**	−3.61	−21.46	9.9					**08-06-15**	−3.17	−3.80	9.4
**25-07-15**	−5.60	−32.87	6.7					**09-06-15**	−2.56	−7.88	14.3
**26-07-15**	−14.89	−116.29	2.1					**10-06-15**	−3.26	−10.43	2.3
**27-07-15**	−11.88	−91.69	1.7					**12-06-15**	−3.14	−14.92	6.6
**28-07-15**	−11.79	−89.74	6.3					**16-06-15**	−4.61	−26.53	3.3
**29-07-15**	−9.58	−66.45	22.2					**25-06-15**	−8.48	−58.67	3.8
**30-07-15**	−11.80	−79.34	7.7					**02-07-15**	−2.79	−11.54	0.8
**31-07-15**	−17.66	−117.45	8.5					**06-07-15**	−4.83	−25.23	1.5
**01-08-15**	−14.99	−97.73	14.0					**15-07-15**	−4.68	−30.64	25.0
**03-08-15**	−8.47	−53.49	2.2					**20-07-15**	−12.09	−89.46	5.3
**12-08-15**	−5.76	−27.84	0.8					**21-07-15**	−10.69	−82.35	6.7
**15-08-15**	−5.90	−30.72	4.0					**10-08-15**	−5.84	−37.86	0.5
**17-08-15**	−5.35	−26.67	1.4					**12-08-15**	−10.30	−69.16	3.8
**21-08-15**	−2.19	−4.87	2.6					**13-08-15**	−6.78	−45.06	1.8
**29-08-15**	−3.75	−17.03	2.2					**14-08-15**	−5.67	−36.12	0.4
**30-08-15**	−4.90	−26.18	2.1					**17-08-15**	−5.25	−34.39	8.2
**04-09-15**	−0.57	6.13	5.0					**19-08-15**	−8.54	−55.45	8.6
**15-09-15**	−6.83	−43.79	3.0					**20-08-15**	−5.49	−36.08	9.4
**16-09-15**	−6.59	−41.92	1.1					**21-08-15**	−8.75	−57.93	1.0
**20-09-15**	−7.53	−56.23	9.4					**25-08-15**	−8.71	−62.02	1.1
**23-09-15**	−8.54	−57.83	17.1					**28-08-15**	−5.83	−37.16	1.0
**02-10-15**	−3.47	−20.92	0.9					**31-08-15**	−3.88	−24.07	5.3
**03-10-15**	−1.70	−7.39	0.3					**01-09-15**	−8.63	−60.14	3.7
								**04-09-15**	−8.11	−62.12	5.0
								**07-09-15**	−4.70	−26.34	1.1
								**21-09-15**	−9.66	−74.33	0.9
								**22-09-15**	−8.08	−60.78	4.3
								**23-09-15**	−8.95	−63.19	17.4
								**24-09-15**	−7.47	−59.17	NA
								**25-09-15**	−8.04	−60.66	NA

In general notation, the isotopic ratios are represented as a delta (δ) value;

δ = (R_sample_/R_VSMOW_ −1) × 10^3^ in per mill (‰)

where, R_sample_ is the ratio of the abundances of the heavier (^18^O or D) to lighter (^16^O or H) isotope in the sample, and R_VSMOW_ is an international standard known as the Vienna Standard Mean Ocean Water (VSMOW) [9].

Example: δ^18^O = {[(^18^O/^16^O) _sample_/(^18^O/^16^O) _VSMOW_] – 1} x 1000‰

## 2 Experimental design, materials, and methods

### 2.1 Sampling method

To collect rainwater samples, a 2-L plastic bottle fitted with a 20.3 cm diameter funnel was used. A tube was attached to the tip of the funnel that touched the bottom of the plastic bottle that helped reduce the exposed surface area, in turn, evaporation. The collected rainwater was converted to rain rate (mm/day) using a calibration equation (available in IAEA/GNIP Precipitation Sampling Guide, v2.02 September 2014). The rainwater samples were transferred to leak-proof plastic bottle and then shipped from respective sites to Indian Institute of Tropical Meteorology (IITM), Pune for isotopic analysis.

### 2.2 Isotopic analysis (δ^18^O & δ^2^H)

**Fig. 1:**
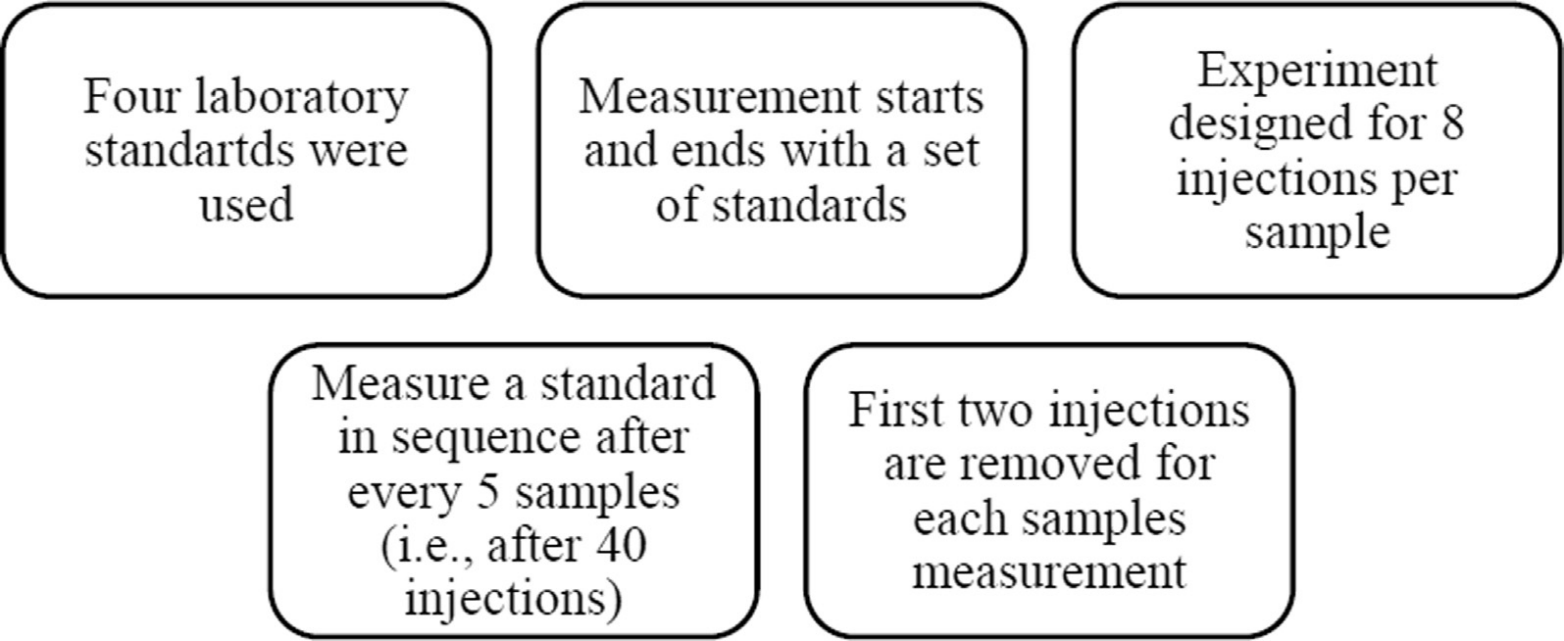
Standards and sample sequence strategy for the isotopic measurement using LWIA.

The LGR's patented liquid water isotope analyzer (LWIA) coupled with a liquid auto-sampler was used for simultaneous measurements of δ^18^O and δ^2^H ratios in water samples. The working of LWIA can be described as follows: all water samples and calibrated laboratory standards are generally kept into a 2mL vials capped with pre-sealed silicone septa. The auto-sampler is used to inject the water sample from the sample tray using a 1.2μL syringe to an injector block (vaporization chamber) heated at 70 °C which is connected to the analyzer. A Teflon tube is connected via an installed filter at the outlet of the tube to the pre-evacuated (using a pump) optical cavity to transfer the vaporized water sample. The memory of the previous sample gas is removed through a vacuum pump and then dry air is passed through the cavity. In addition, initial two injections used to be removed during post-processing to minimize the memory effect. The followed sequence of laboratory standards and samples for the isotopic measurement are described in Fig. 1.

The LWIA post-processing software interface allows the user to enter sample information for analyses. The analyzer measures concentrations of the individual isotopologues of H_2_O and calculates absolute ratios. Therefore, to convert isotopic ratios relative to the VSMOW standard, the absolute ratios are recalculated using calibrated laboratory working standards (Table 2) and their ratios relative to VSMOW. The overall analytical precision obtained for δ^18^O (δ^2^H) was about 0.1‰ (<1‰).

**Table 2:** Laboratory standards used for the isotopic measurement. The names are coded in order to identify the source and properties of the standards, such as QD_2_O - double distilled water prepared in a quartz distillation plant; En-stands for isotopically enriched water.

S. No.	Laboratory Standard	δ^18^O (‰)	δD (‰)
1	QD_2_O-B	−4.39	−29.30
2	IITM-A	−2.04	−10.08
3	IITM-En	3.71	11.03
4	IITM-En2	7.97	26.37
